# ORFI genetic polymorphisms in geographically distinct HTLV-1 infections

**DOI:** 10.1186/1742-4690-12-S1-P55

**Published:** 2015-08-28

**Authors:** Fernanda Khouri Barreto, Ricardo Khouri, Filipe Ferreira de Almeida Rego, Maria Fernanda de Castro-Amarante, Izabela Bialuk, Cynthia A Pise-Masison, Bernardo Galvão-Castro, Antoine Gessain, Steven Jacobson, Genoveffa Franchini, Luiz Carlos Alcantara

**Affiliations:** 1 Centro de Pesquisa Gonçalo Moniz-Fiocruz, Salvador, Bahia, Brazil; 2 Escola Bahiana de Medicina e Saúde Pública, Salvador, Bahia, Brazil; 3 Animal Models and Retroviral Vaccines Section, National Cancer Institute, Bethesda, USA; 4 Department of General and Experimental Pathology, Medical University of Białystok, Poland; 5 Unité d'Epidémiologie et Physiopathologie des Virus Oncogènes, Département de Virologie, Batiment Lwoff, Institut Pasteur, Paris, France; 6 Viral Immunology Section, Neuroimmunology Branch, National Institute of Neurological Disorders and Stroke, Bethesda, USA

## 

The region known as pX in the 3’ end of the human T-cell leukemia/lymphoma virus type-1 (HTLV-1) genome contains four overlapping open reading frames (ORF) that encode regulatory proteins. The expression of ORF-I produces the protein p12 which can be cleavage resulting in the p8 protein. These proteins interfere with the ability of a biologically active of HTLV-1, influencing at the virus infectivity and persistence. Here, we evaluated whether natural mutations in HTLV-1 ORF-I can influence the proviral load and clinical manifestation of HAM/TSP. For that, we analyzed 1530 HTLV-1 ORF-I sequences from different clones of 156 patients with negative or positive diagnosis for HAM/TSP and demonstrated that some mutations may be associated with the outcome of HAM/TSP (C39R, L40F, P45L, S69G and R88K) or with proviral load (P34L and F61L). We further examined the presence of mutations in motifs of HBZ and observed that P45L mutation is located within the HBZ nuclear localization signal and was found more frequently between patients with HAM/TSP and high proviral load. This results suggest that some HTLV-1 ORF-I mutations may be associated with the development of HAM/TSP and the proviral load levels.

